# Predictive Outcome Modeling of Preoperative Clinical Symptoms and Electrodiagnostic Data in Tarsal Tunnel Surgery

**DOI:** 10.1055/s-0041-1731747

**Published:** 2021-07-27

**Authors:** Geoffrey K. Seidel, Salma Al Jamal, Eric Weidert, Frederick Carington, Michael T. Andary, Scott R. Millis, Brian G. Loder

**Affiliations:** 1 Department of Physical Medicine and Rehabilitation, Wayne State University School of Medicine, Michigan State University College of Osteopathic Medicine, Henry Ford Macomb Hospital, Clinton Township, Michigan, United States; 2 Department of Obstetrics and Gynecology, Advocate Aurora Health, Aurora Healthcare, Milwaukee, Wisconsin, United States; 3 Department of Physical Medicine and Rehabilitation, College of Osteopathic Medicine, Sparrow Hospital, Michigan State University, McLaren Hospital of Greater Lansing, Lansing, Michigan, United States; 4 Department of Physical Medicine and Rehabilitation, School of Medicine, Wayne State University, Detroit, Michigan, United States; 5 Department of Orthopaedics, College of Osteopathic Medicine, Henry Ford Macomb Hospital, Michigan State University, Clinton Twp., Michigan, United States

**Keywords:** tarsal tunnel syndrome, nerve conduction, medial plantar nerve, lateral plantar nerve, surgery, outcome modeling

## Abstract

**Background** The relationship between tarsal tunnel syndrome (TTS), electrodiagnostic (Edx) findings, and surgical outcome is unknown. Analysis of TTS surgical release outcome patient satisfaction and comparison to Edx nerve conduction studies (NCSs) is important to improve outcome prediction when deciding who would benefit from TTS release.

**Methods** Retrospective study of 90 patients over 7 years that had tarsal tunnel (TT) release surgery with outcome rating and preoperative tibial NCS. Overall, 64 patients met study inclusion criteria with enough NCS data to be classified into one of the following three groups: (1) probable TTS, (2) peripheral polyneuropathy, or (3) normal. Most patients had preoperative clinical provocative testing including diagnostic tibial nerve injection, tibial Phalen's sign, and/or Tinel's sign and complaints of plantar tibial neuropathic symptoms. Outcome measure was percentage of patient improvement report at surgical follow-up visit.

**Results** Patient-reported improvement was 92% in the probable TTS group ( *n* = 41) and 77% of the non-TTS group ( *n* = 23). Multivariate modeling revealed that three out of eight variables predicted improvement from surgical release, NCS consistent with TTS ( *p* = 0.04), neuropathic symptoms ( *p* = 0.045), and absent Phalen's test ( *p* = 0.001). The *R* ^2^ was 0.21 which is a robust result for this outcome measurement process.

**Conclusion** The best predictors of improvement in patients with TTS release were found in patients that had preoperative Edx evidence of tibial neuropathy in the TT and tibial nerve plantar symptoms. Determining what factors predict surgical outcome will require prospective evaluation and evaluation of patients with other nonsurgical modalities.

## Introduction

Tarsal tunnel syndrome (TTS) is difficult to diagnose without universal agreement on the diagnostic criteria. TTS is usually associated with pain, numbness, and tingling on the bottom of the foot often involving the heel, plantar surface of the foot, as well as the toes, in the tibial nerve distribution; calcaneal, medial plantar (MP), and lateral plantar (LP) branches. TTS often has diffuse, poorly localizable, plantar symptoms and physical examination findings fail to confirm tibial nerve compression. 1 Electrodiagnostic (Edx) evaluation assists in confirmation of neurophysiologic abnormalities within the tarsal tunnel (TT). 2 3 Edx studies have documented measurable changes of tibial nerve conduction studies (NCSs) with good outcomes following surgical decompression. 2 4 5 6 7 These studies had small patient numbers and primarily assessed tibial motor and MP and LP sensory nerve action potentials (SNAPs) but not mixed nerve action potentials (MNAPs). MNAPs (historically labeled compound nerve action potentials or CNAPs) measure both sensory and motor action potentials along the MP and LP nerve branches of the tibial nerve.

Preoperative TTS surgical decompression decisions are often based on clinical symptoms and physical examination findings alone without Edx testing. 1 8 Furthermore, when preoperative Edx testing is completed, motor testing is the only modality assessed. Tibial motor NCSs are the most studied method to evaluate TTS but MP and LP SNAPs are more sensitive than the motor studies in the diagnosis of TTS. 1 2 3 8 It is currently recommended that both tibial motor and sensory NCS to be performed when evaluating a patient for TTS. 3 MP and LP SNAPs have been obtained by stimulation of a single toe and orthodromic proximal recording across the TT but these studies are technically difficult and do not evaluate all sensory MP and LP nerve fibers.

The purpose of this study was to perform retrospective analysis of patients who had TT releases and preoperative NCS data to determine which clinical and Edx data are predictive of outcome. Our study utilized a similar MP and LP MNAP study technique described in several publications. 9 10 11 12 MNAPs are considered superior in TTS assessment because the NCS includes a larger number of sensory and motor axons contributing to the measured waveform which is considered the basis for increased specificity over SNAPs for TTS diagnosis. 11 Our study used the most up to date TTS Edx evidence combined with one of the largest subject outcome assessments in published reports. This study is hoped to bring further clarity to both surgical and electrodiagnostic practitioners when dealing with TTS.

## Materials and Methods

Surgeries and Edx studies were performed in the routine course of practice in the Health Insurance Portability and Accountability Act (HIPAA) compliant practices with institutional review board (IRB) approved retrospective data analysis. This study was a collaborative effort between two university settings. All surgical interventions were performed by a board-certified (the American Board of Foot and Ankle Surgery) podiatrist (B.G.L.) with the same technique. The surgeon decided which patients required surgical intervention based on clinical symptoms and Edx studies. The Edx studies were available to the surgeon; however, we are not able to quantify how much the Edx results influenced the decision to perform surgery. Some patients underwent surgery with normal Edx studies. Presumably, there were abnormal Edx tests where the surgeon or patient decided against surgery. We do not have access to that group. The surgical procedure was performed in the surgical theater under general anesthesia following appropriate sterilization protocols. The posterior tibial nerve release was approached through a curvilinear incision in the posterior ankle compartment taking care to release the retinaculum completely both proximally and distally. The dissection was performed with a hands-off technique to avoid any trauma to the nerve but with care to facilitate complete decompression of MP and LP, as well as the calcaneal nerve branches.

The surgeon provided a list of patients with TT releases performed. The medical records of these patients were reviewed for clinical history including sex, age, body mass index (BMI), surgical side, Edx study date, surgery date, podiatry follow-up date, diabetes mellitus, the presence or absence of tibial-nerve-specific neurogenic symptoms in the heel and/or bottom of the foot and toes, tibial nerve block results, tibial nerve Tinel's sign, and Phalen's sign. A positive Tinel's sign was defined as neurogenic dysesthesias radiating distally into the plantar aspect of the foot with percussion over the tibial nerve. A positive Phalen's sign was either a recreation of clinical symptoms or tibial nerve dysesthesias in the plantar aspect of the foot when the ankle was passively, maximally everted and held in this position. Tibial Tinel's and Phalen's clinical tests were performed and interpreted by the podiatrist. Surgical outcome data were based on patient-report percent postoperative improvement at podiatry follow-up office visit.

**Fig. 1:**
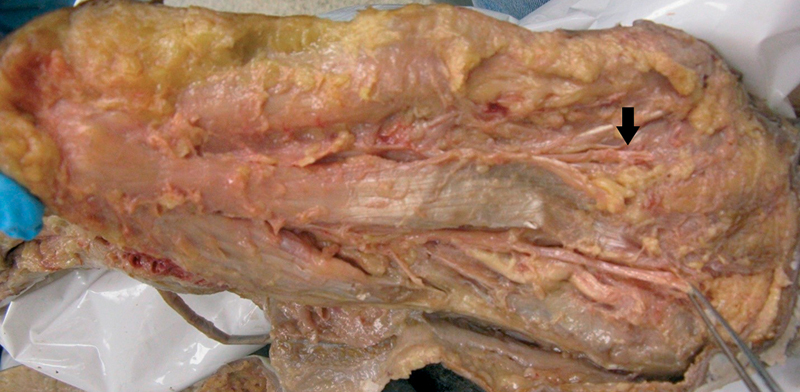
Foot plantar dissection with forceps holding medial plantar nerve and arrow marking lateral plantar nerve. Nerve stimulation performed at arrow location.

**Fig. 2:**
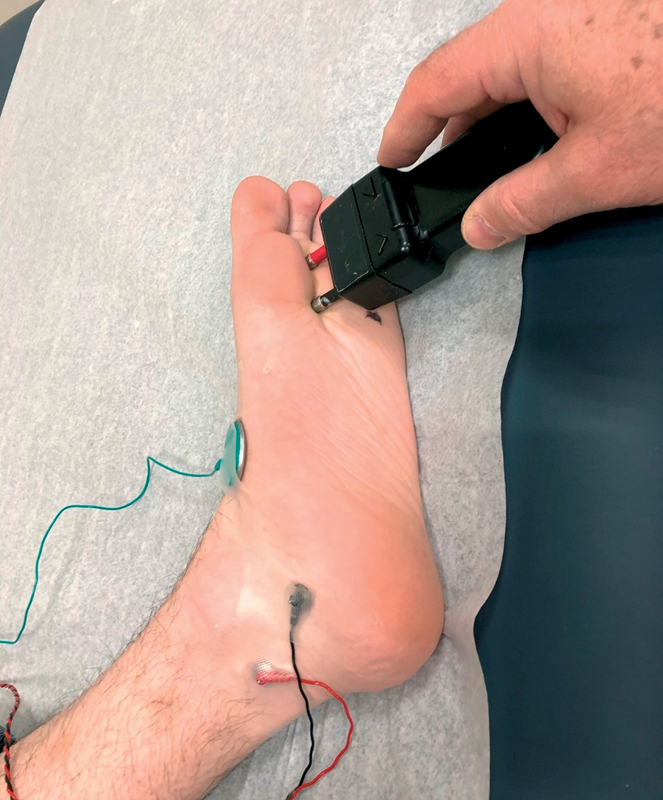
Cathode (black) stimulation of medial plantar MNAP just proximal to the metatarsal heads between the first and second metatarsals with E1 (black) 14-cm proximal across the tarsal tunnel on the tibial nerve. Lateral plantar stimulation is achieved by moving the cathode to the mark between the fourth and fifth metatarsals with the same E1 distance and setup. MNAP, mixed nerve action potential.

The same board-certified electromyographer (G.K.S.) performed all studies with foot temperature ≥ 29°C. Standard procedures for Edx testing were performed on a Cadwell's Sierra Wave 2. NCS included sural (14 cm) and/or superficial fibular (12 cm) SNAPs, fibular (9 cm) and tibial (8 cm) compound motor action potentials (CMAPs), as well as MP and LP MNAPs. 13 14 15 16 17 MNAP stimulation was performed on the MP with the recording E1 electrode placed over the tibial nerve proximal to the TT at a distance of 14 cm. A cloth tape was used to measure the distance starting at a location between the base of the first and second metatarsal heads where stimulation was performed across the TT along the tibial nerve. The LP setup was the same at a distance of 14 cm with the measurement starting at a location between the base of the fourth and fifth metatarsal heads across the TT along the tibial nerve. This technique was modified slightly from that described in prior studies, 9 10 11 12 as the MP and LP MNAPs were stimulated more distally just proximal to the metatarsal heads to avoid stimulating through the plantar fascia and musculature that can often interfere with MNAPs. This modified technique was based on cadaver dissections ( Fig. 1 ) and technical ease of obtaining MNAPs during Edx evaluation ( Fig. 2 ).

Data were recorded in tabular form including for sensory peak latency (PL) and amplitude measured from baseline to peak, as well as screenshots of waveforms. Data were recorded in tabular form for CMAPs included distal latency (DL), amplitude and velocity, as well as screenshots of waveforms. SNAP and MNAP waveforms were averaged twice to thrice for optimal report presentation. Only the number of nerve stimulations required to assess patients for routine Edx purposes were performed. The minimum number of NCS was performed on each patient to establish an Edx diagnosis and thus every possible NCS was not performed on every patient.

Tibial neuropathy in the TT consistent with the diagnosis of TTS Edx criteria was defined prior to the initiation of the study. A diagnosis of TTS required tibial motor DL ≥2 ms beyond fibular motor DL and/or reduced amplitude and including MP and LP MNAP PL ≥1 ms slower than the sural. This category required normal sensory (sural and/or superficial fibular) and fibular CMAP studies. Possible TT (PTT) was defined as abnormal MP and LP MNAPs if PL was delayed (≥1.0 ms slower than sural) or absent response in the presence of a normal sural SNAP, as well as both tibial and fibular CMAPs. Deidentified, blinded to clinical symptoms, tabular NCS data with waveforms for reference were presented for interpretation to an electromyographer (M.T.A.) at another university setting who verified data and grouped data in categories of TTS, PTT, peripheral polyneuropathy (PPN), and Normal (Norm). PPN was defined as delayed PL and/or reduced amplitude or absent sural SNAP, delayed PL, and/or reduced amplitude or absent tibial and fibular CMAP with corresponding MP and LP MNAPs. Norm was defined as all NCSs in normal range. Standard electromyography needle examination performed on all patients included first dorsal interosseous pedis, medial and lateral gastrocnemii, bicep short head, semimembranosus, vastus medialis, gluteus medius, and lumbar paraspinals. Lumbosacral radiculopathy was not diagnosed in any of the study participants (positive waves/fibrillations in muscles proximal to the foot). The reviewer erred on the side of strict Edx criteria for category designation. The reviewer was also blinded to Edx interpretation and surgical outcomes.

Inclusion criteria: patients sequentially from 2010 to 2017 initially identified by the surgeon who had TT surgical decompression ( *n* = 104). Those patients who were lost to follow-up ( *n* = 8) or had no surgical outcome recorded ( *n* = 6) were not included (total not included *n* = 14 (14/104 = 13%) in the study. The Edx study data for the remaining ninety patients ( *n* = 90) were subject to exclusion criteria.

Exclusion criteria: participants with inadequate tibial nerve NCS assessment by other electromyographers in different Edx facilities ( *n* = 18). The 20% (18/90) excluded studies had only tibial CMAPs reported. The remaining study participants ( *N* = 72) all had Edx evaluation by the same electromyographer (G.K.S.) and had been referred by the surgeon (B.G.L.) for either unilateral or bilateral lower extremity Edx evaluation. Those patients with bilateral lower extremity Edx evaluation were treated as single individuals by eliminating one limb randomly to decrease bias ( *n* = 8). Statistical analysis was performed on the remaining 64 patients.

### Statistical Analysis

Descriptive statistics including age, sex, and side studied and NCS parameters were performed. Predictive statistical modeling including nerve conduction data, clinical signs, and symptoms was performed. Statistical analysis was conducted with StataCorp. 2019. Stata Statistical Software: Release 16 (StataCorp LLC; College Station, TX).

Multivariable modeling was used to examine which variables were significant predictors of patient-rated improvement. We initially considered employing standard multiple regression analysis. However, the patient-rated improvement outcome variable is measured as a percentage or proportional rate of improvement. As such, the improvement variable can take on values ≥0 and ≤1. Multiple regression assumes an unbounded outcome variable. In fact, when we fit a multiple regression model, significant heteroskedasticity was found (Breusch–Pagan test, *p* = 0.002). Thus, we opted to use a fractional response regression model with robust standard errors. Improvement outcome modeling was first performed on TTS group rating, selected NCS variables and clinical symptoms ( Table 4 ). The predictor variables entered simultaneously into the model were selected to evaluate motor and sensory latencies and clinical signs as predictors. The use of MP and LP MNAP amplitudes was not considered because of the large number of absent responses in the probable TTS (ProbTTS) and the PPN groups. In modeling, absent responses are automatically discarded as no data thus not a good variable to include in modeling. There was no indication of substantial collinearity among this set of predictor variables: all variance inflation factors (VIF) were less than 3.00. High collinearity can cause problems with fitting and interpreting predictions of outcome in a model which was not observed in the analysis of data in this study.

## Results

Overall, 64 patients with TTS surgical decompression and Edx NCS data descriptive group statistics are presented in Table 1 . Among them, 81% were female with equal right/left releases. No patients had radiculopathy. Mean office follow-up was 487 days (standard deviation [SD] = 535, range: 27–2,286).

**Table 1:** Patient descriptive demographics and preoperative clinical examination observations

Variable	Patients
Female ( *n/N* , %)	52/64, 81.3
Age, mean years ( ± SD)	50.9, 13.6
BMI, mean kg/m ^2^ ( ± SD)	30.2, 6.8
DM type 2 ( *n/N* , %)	8/64, 12.5
Surgical side	
Right ( *n/N* , %)	32/64, 50
Left ( *n/N* , %)	32/64, 50
Positive nerve block ( *n/N* , %)	40/63, 63.5
Positive Tinel's sign ( *n/N* , %)	50/52, 96.2
Positive Phalen's sign ( *n/N* , %)	33/40, 82.5
Nerve symptoms ( *n/N* , %)	59/63, 93.7

Abbreviations: BMI, body mass index; DM, diabetes mellitus; SD, standard deviation.

Edx category rating designation was based on the totality of NCS data resulted in stratification TTS ( *n* = 4), PTT ( *n* = 37), PPN ( *n* = 14), and Norm ( *n* = 9; Table 2 ). There was no significant difference in BMI between the groups. Males and females were proportionally represented in each group. Postoperative improvement was robust in TTS (93.8%) and PTT (92.0%), but improvements were also noted in PPN (82.5% and Norm, 67.8%). NCS group descriptive data are presented in Table 3 .

**Table 2:** Stratified groups based on age, BMI and percentage of improvement patient rating, delta motor– and delta sensory–derived variables following surgery

	*N*	Age in y (mean, SD)	BMI in kg/m ^2^ (mean, SD)	% Improve (mean, SD)	DMT in ms (mean, SD)	DSN in ms (mean, SD)
TTS	4	49.5, 18.4	26.9, 5.1	93.8, 12.5	1.9, 0.2	7.9, 5.4
PTT	37	51.2, 11.9	30.7, 7.3	92.0, 17.9	1.0, 0.9	9.3, 5.3
PPN	14	58.6, 10.2	32.3, 5.3	82.5, 28.7	0.5, 0.7	−1.0, 4.1
Norm	9	38.3, 15.4	26.0, 6.0	67.8, 36.7	−0.1, 0.9	7.3, 6.4
Total	64	50.9, 13.6	30.2, 6.8	86.6, 24.6	0.8, 1.0	6.7, 6.6

Abbreviations: BMI, body mass index; DMT, tibial motor minus fibular motor distal latency; DSN, sural sensory amplitude minus (medial + lateral plantar amplitudes)/2; Norm, electrodiagnostic normal; PPN, electrodiagnostic peripheral polyneuropathy; PTT, electrodiagnostic possible tarsal tunnel syndrome; SD, standard deviation; TTS, electrodiagnostic tarsal tunnel syndrome.

**Table 3:** Nerve conduction study data stratified by group designation (TT, PTT, PPN, and Norm)

	TT *N* = 4				PTT *N* = 37				PPN *N* = 14			Norm *N* = 9				
	*n*	Mean/SD	Min/Max	NR (%)	*n*	Mean/SD	Min/Max	NR (%)	*n*	Mean/SD	Min/Max	NR (%)	*n*	Mean/SD	Min/Max	NR (%)
Sural																
PL (ms)	3	3.9/0.1	3.8/4	0	35	3.8/0.5	2.5/5.2	0	1	4.5		93	9	3.7/0.3	3.3/4.1	0
Amp (uV)	3	10.5/1.5	9.0/12.0	0	35	10.6/4.6	5.0/22.0	0	1	5.6		93	9	15.7/6.5	10.0/25.7	0
Superficial fibular																
PL (ms)	1	3.8		0	5	3.7/0.1	3.5/3.8	0	5			100	1	3.6		0
Amp (uV)	1	14		0	5	8.9/4.4	6.0/16.7	0	5			100	1	10.6		0
Fibular motor																
DL (ms)	4	5.0/0.5	4.3/5.4	0	31	4.8/0.6	3.6/5.9	0	11	4.9/0.6	3.8/6.0	20	8	5.0/0.8	4/6.2	13
Amp (mV)	4	2.7/0.8	1.6/0.5	0	31	4.3/1.5	1.4/7.0	0	11	3.8/2.4	1.6/10.1	20	8	4.9/2.0	1.9/7.7	13
CV (m/s)	4	46/2.9	43/9	0	31	46/4.2	37.0/60.0	0	11	46/5.3	39/56	20	8	48/4.7	40/53	13
Med plantar																
PL (ms)	4			100	8	6.2/1.3	3.9/8.4	78	3	5.9/0.5	5.3/6.3	79	9	3.7/0.4	3.3/4.5	0
Amp (uV)	4			100	8	6.1/3.5	3.3/12.3	78	3	8.9/5.1	5.7/14.8	79	9	7.8/3.2	3.0/12.1	0
Lat plantar																
PL (ms)	4			100	4	5.4/1.1	4.1/6.7	89	1	4.9		93	8	4.0/1.0	3.4/6.3	13
Amp (uV)	4			100	4	5.3/0.2	5.0/5.5	89	1	12.3		93	8	9.1/3.0	4.0/12.3	13
Tibial motor																
DL (ms)	4	6.9/0.4	6.4/7.3	0	37	5.6/1.0	3.3/7.8	0	12	5.5/0.9	4.5/7.4	14	9	4.8/0.8	4.1/6.4	0
Amp (mV)	4	10.4/2.6	6.7/12.5	0	37	7.6/3.6	0.6/15.4	0	12	6.6/4.5	1.1/14.8	14	9	7.8/2.8	2.9/11.3	0
CV (m/s)	4	45/1.0	43/45	0	35	47/5.9	38/59	2/5.0	12	44/4.6	35/49	14	9	48/4.6	41/55	0

Abbreviations: Amp, amplitude; CV, conduction velocity; DL, distal onset latency; Max, maximum; Min, minimum; Norm, normal; NR, not reported; PL, peak latency; PPN, peripheral polyneuropathy; PTT, possible tarsal tunnel; SD, standard deviation; TT, tarsal tunnel.

Note: Group numbers ( *N* ), Mean, Range descriptive statistics are provided for each nerve, as well as absent response (NR).

Due to the small TTS group ( *N* ), this group was combined with the PTT group for analysis purposes ( *n* = 41). This combined group was considered ProbTTS. Groups PPN and Norm were considered non-TTS and combined for analysis ( *n* = 23). Mean improvement following TT decompression in the ProbTTS group (92.2%) was greater than the non-TTS group (76.7%). Independent samples test for equality of variance equal variances not assumed was significant ( *p* = 0.0001), *t* -test for equality of means equal variance not assumed was significant ( *p* = 0.041).

The small number of cases that met the rigid requirement of 2-ms difference between fibular DL (FMDL) and tibial DL (TMDL) was designated delta motor (DMT) and may have excluded patients with clinically significant TTS. There was a significant DMT difference between TTS-PPN ( *p* = 0.034), TTS-Norm ( *p* = 0.002), and PTT-Norm ( *p* = 0.013). There was no significant DMT difference between TTS-PTT and PTT-PPN. DMT was then stratified into three groups based on time: 0 to 1, 1 to 2, and >2 ms to determine if DMT was a predictor of outcome. ProbTTS had a mean DMT of 1.1 ms (SD = 0.9), while non-TTS was 0.18 ms (SD = 0.9). There was a significant difference between ProbTTS versus non-TTS DMT by the *t* -test equal variances assumed two-tailed ( *p* = 0.002). There was no significant difference between the DMT groups when considering patient improvement rating.

The ProbTTS group had absent MP (78%) and LP (89%) responses considered consistent with TTS ( Table 3 ). Peak latencies for MP and LP MNAP absent response data could not be utilized to calculate mean descriptive statistics; however, amplitudes of 0 considered clinically meaningful were able to be described statistically in a derived variable. Delta sensory (DSN) was calculated by subtracting the sum of the MP and LP amplitudes divided by two from the sural amplitude to facilitate analysis ( Table 2 ). There was a significant DSN difference between PTT-PPN ( *p* = 0.0001), TTS-PPN ( *p* = 0.02), and PPN-Norm ( *p* = 0.003). The following groups failed to demonstrate a significant difference ( *p* < 0.05): TTS-PTT, PTT-Norm, and TTS-Norm.

Outcome predictive modeling: the omnibus test for the overall regression model was statistically significant, Wald's Chi-square = 1,604, *p* < 0.001. Of the eight predictors, three variables were statistically significant predictors of patient-rated improvement ( Table 4 ): ProbTTS ( *p* = 0.04), nerve symptoms ( *p* = 0.045), and absence of Phalen's sign ( *p* = 0.001). Specifically, the ProbTTS group, nerve symptoms, and the absence of Phalen's sign were associated with greater patient-rated improvement. Medial plantar peak latency (MPPL) and nerve block showed a trend toward significance with higher MPPL and nerve block being associated with greater improvement. The pseudo *R* ^2^ was 0.21 which is reasonable in light of the multifactorial nature of human attitude research. Additional significant predictors may have been absent in this model.

**Table 4:** Improvement outcome regression modeling group rating, selected nerve conduction study parameters, and clinical examination observations

		Robust SE				
Improvement	Coefficient		*z*	*p* > | *z* |	[95% CI]	
ProbTTS	1.687	0.820	2.06	0.040	0.078	3.296
MPPL	0.378	0.224	1.69	0.092	−0.061	0.817
LPPL	−0.276	0.246	−1.12	0.262	−0.760	0.206
TMDL	−0.413	0.300	−1.38	0.169	−1.001	0.175
NBlock	1.248	0.689	1.81	0.070	−0.102	2.599
Tinel's sign	0.061	1.491	0.04	0.967	−2.862	2.985
Phalen's sign	−16.06	0.718	−22.3	0.001	−17.47	−14.65
NSymp	1.550	0.773	2.00	0.045	0.034	3.067
Constant	16.51	2.561	6.45	0.00	11.499	21.538

Abbreviations: CI, confidence interval; LPPL, lateral plantar peak latency; MPPL, medial plantar peak latency; NBlock, tibial nerve block; NSymp, tibial plantar neurogenic symptoms; Phalen's sign, tibial Phalen's result; ProbTTS, electrodiagnostic probable tarsal tunnel syndrome; SE, standard error; Tinel's sign, tibial nerve Tinel's result; TMDL, tibial motor distal latency.

A second improvement outcome modeling was performed with ProbTTS, tibial nerve symptoms, and NCS data (including MNAP amplitudes; Table 5 ). MP and LP MNAP absent amplitude responses were designated zero. The predictor variables entered simultaneously into the model included ProbTTS, DMT, tibial nerve symptoms, MPPL, LPPL, and DSN. The omnibus test for the overall regression model was statistically significant, Wald's Chi-square = 37.78, *p* < 0.00001. Of the six predictors, two variables were statistically significant predictors of patient-rated improvement: ProbTTS ( *p* = 0.009) and nerve symptoms ( *p* = 0.002). The derived variables of DMT and DSN were not predictive of outcome.

**Table 5:** Improvement outcome regression modeling group rating (ProbTTS), nerve symptoms, and nerve conduction study data

		Robust SE				
Improvement	Coefficient		*z*	*p* > *z*	[95% CI]	
ProbTTS	1.255	0.483	2.60	0.009	0.308	2.202
Delta motor	0.236	0.162	1.46	0.145	−0.081	0.555
NSymp	1.763	0.581	3.03	0.002	0.624	2.903
MPPL	0.180	0.149	1.21	0.228	−0.111	0.473
LPPL	−0.187	0.198	−0.94	0.346	−0.577	0.202
Delta sensory	−0.015	0.053	−0.29	0.768	−0.204	0.088
Constant	−0.205	0.708	−0.29	0.771	−1.593	1.182

Abbreviations: CI, confidence interval; Delta Motor, tibial motor minus fibular motor distal latency; Delta sensory, sural sensory amplitude minus (medial + lateral plantar amplitudes)/2; LPPL, lateral plantar peak latency; MPPL, medial plantar peak latency; NSymp, tibial plantar neurogenic symptoms; ProbTTS, electrodiagnostic probable tarsal tunnel syndrome; SE, standard error.

## Discussion

This retrospective study of patients with TT surgical release procedures, follow-up outcome patient rating, as well as preoperative Edx NCSs, focused on outcome modeling. Patients with Edx ProbTTS or tibial plantar neurologic symptoms were more likely to report postoperative improvement in the presented study. While there were some predating studies that offer similar criteria and Edx studies to show evidence of probable TTS, 10 11 postoperative follow-up data, 2 3 4 5 6 18 or large sample sizes, 4 5 18 none seem to have all three as we demonstrated in this study. We only found one other study that reported surgical outcomes and MNAP studies but NCS data and correlation to outcome was not reported. 19 We found no case series that reported both preoperative MNAP data and postoperative outcome. TTS is often difficult to diagnose on clinical grounds alone and Edx studies are recommended to document abnormal tibial nerve function to improve the odds of a good surgical outcome. 1 8 18 This study demonstrated that those patients with Edx evidence of TTS (the ProbTTS group) had greater improvement in postoperative outcome reports than those patients who had Norm or PPN NCS results. Those patients with Norm or PPN Edx studies also reported postoperative improvement, but the degree of improvement was significantly less in these groups ( Table 2 ). This finding suggests that patients with a combination of NCS evidence and clinical symptoms consistent with TTS and will have better outcomes. The mean surgical office reevaluation of 420 days reported represents sustained postoperative improvement. However, it was not possible to determine the time course of symptom or Edx improvement.

Provocative clinical signs, including Tinel's and Phalen's signs, were not predictive of postoperative clinical improvement. In fact, a positive Phalen's sign pointed to a worse surgical outcome in this study. It was unexpected that a positive Phalen's sign would predict a worse outcome. A possible explanation is that only 63% of patients had documented Phalen's testing and this may have impacted this result. It is also possible that the Tinel's sign done in this manner can be false positive and mislead clinicians. A positive tibial nerve block neared statistical significance ( *p* = 0.07) as a predictor of surgical outcome and may be a good clinical predictor of outcome. Tibial nerve symptoms and provocative signs are clinical tools that are suggestive of a clinical TTS diagnosis, but our study supports the value of objective Edx assessment and consideration of a tibial nerve block to further assess which patients could potentially benefit from surgical release.

We were surprised that no TTS patients in this study had denervation evident in the first dorsal interosseous pedis. We are unsure why. It is possible that our study group did not include advanced or severe cases of TTS with denervation evident. We still think that, in cases of TTS, electromyographers should perform needle assessment in abductor hallucis, first dorsal interosseous pedis or other tibial innervated foot muscles, and compare them to the fibular innervated extensor digitorum brevis.

This study documents significant improvement in patients with abnormal or absent MP and LP MNAP results with a normal tibial CMAP. The authors conclude that requiring an abnormality in the tibial CMAP, as well as MP and LP MNAPs, maybe too strict of a criterion to document Edx TTS. The totality of the NCS data interpreted as consistent with Edx TTS was the most predictive of surgical outcome patient rating ( *p* = 0.04) of all variables evaluated. The only solitary Edx NCS finding that approached significance as the most predictive of outcome rating was the MPPL in the predictive model ( *p* = 0.09). However, it is recognized that the predictive model based on latencies only (TMDL, MP, and LP PLs) failed to explain all variants in outcome. Latencies were initially chosen for the predictive model because it was felt these variables would be most predictive of outcome. Based on the limits of predictive modeling, post hoc expansion of the predictive modeling variable list beyond eight variables (to include amplitudes) with preoperative clinical observations was not done based on limits of the study size ( *n* = 64). The absence of MP and/or LP MNAPs was by far the most common abnormality in the Edx ProbTTS group. DMT was evaluated to determine if this variable would explain the limited variance in the predictive model, but the DMT unexpectedly did not significantly predict surgical outcome improvement. The distance utilized for the fibular CMAP study may have impacted predictive modeling as it was 9 cm (while the tibial distance was 8 cm), this may have increased the fibular DL by 12% compared with the tibial thus the DMT may have been closer to 1.4 ms had the distance been exactly equal. The lack of significant difference between the PTT-PPN groups was considered a consequence of the differences which can be observed in DMT in PPN. The failure to observe patient improvement with DMT may be due to difficulty accounting for TMDL premotor potential often observed in TTS that may have artificially shortened TMDL.

Several patients in the PPN group, in this study, improved following surgical release. The reviewer (M.T.A.) may have been too strict in defining Edx criteria to diagnose TTS. There may be patients in the PPN group who also had TTS (TTS superimposed on PPN) which was not recognized based on the strict NCS criteria established prior to the study. In a study of painful diabetic neuropathy with tibial nerve decompression, other authors have observed that tibial motor NCS parameters did demonstrate postoperative improvement; however, MP and LP SNAP or MNAP studies were not performed. 20 The clinical reality is that there are patients with PPN who can have TTS and do benefit from TT release. In an effort to further clarify Edx variables that might explain improvements reported in the PPN group, a post hoc analysis was performed. There were 4 of 14 PPN cases with 100% improvement reports that had a delta motor >1.5 ms with absent MP and LP MNAPs. It is considered that the number in this group was too small to make general conclusions about diagnosing Edx TTS superimposed on PPN. How to better define and categorize this group who would be best suited for surgical decompression would need further study.

The majority of the patients in the Norm Edx group improved following surgical release of the TT. The reviewer (M.T.A.) may have been too strict, again as in the PPN group, in defining Edx criteria to diagnose TTS. It was observed that in five of nine cases with 100% improvement reports, the MP and LP amplitudes were ≤50% of the sural, while the MP and LP peak latencies were normal. Two cases reported >50% postoperative improvement. If only one of the plantar MNAPs (MP or LP) amplitude was ≤50% of the sural with a normal Tibial CMAP. It is possible that a larger sample may reveal that in the presence of otherwise normal NCS the presence of ≤50% MP and LP amplitudes relative to the sural might correlate with improved surgical outcomes.

The biggest difference between the ProbTTS and PPN groups was sural versus MP and LP amplitude difference (the sural amplitude was significantly larger than the MP and LP amplitudes). Thus, DSN was included in a second regression analysis focused on more inclusive NCS variables to determine if these variables individually would be predictive of outcome. Edx group designation and tibial nerve clinical symptoms again best predicted postoperative improvement. The remaining NCS variables were not individually predictive of outcome. This failure of individual NCS variables to predict outcome may be due to multiple factors including the subjective nature of the percent outcome rating variable. We were not able to establish a pattern of NCS data abnormalities that clearly distinguished group designation.

## Limitations and Strengths

The retrospective nature of this study leads to multiple possible weaknesses. This study did not have objective follow-up NCS data to document objective improvement in neurophysiological parameters. Calcaneal NCSs were not performed in this study, so comparison including this distal tibial nerve branch was not possible. Retrospective chart reviews by their nature may have incomplete data available for analysis, as was the case in this study. Patient-percentage outcome rating is a subjective assessment equivalent to patient satisfaction and the postoperative patient rating scores were notable even in PPN and Norm patient groups. We are unsure of how much the NCS data were considered in surgical decision-making to decompress the tibial nerve in the TT as patients with Norm Edx testing did undergo surgical intervention. Patient outcome rating was reported to the surgeon making bias probable.

One of the strengths of the data presented in this study is the large patient number verses prior published reports and the detailed analysis with predictive modeling of the MP and LP MNAPs that have not been previously reported.

The determination of normal versus abnormal in TTS Edx testing is unclear and we are unable to document full evidence-based criteria at this time. Accepting the inherent limitations of this retrospective study, the complicated interplay of often imprecise clinical symptoms and physical examination findings, it is recommended based on this study that Edx testing that be utilized to improve the odds of good surgical decompression outcomes. Based on the data presented in our study, we recommend the Edx criteria for a diagnosis of TTS include delayed or absent MP and/or LP MNAPs with or without abnormal tibial CMAP parameters. In the absence of peripheral neuropathy, NCS abnormalities should be observed in two out of three tibial nerve functions: tibial CMAP, MP, and LP MNAPs. The tibial CMAP DL and/or amplitude could be abnormal. The MP and LP MNAPs may be absent, delayed, or reduced amplitude. The amount of delay or amplitude decrease is unclear. An MP and/or LP MNAP at 1.0-ms PL delay and/or 50% reduced amplitude compared with sural may prove to be significant parameters in TTS Edx diagnosis. Similarly, for relative tibial motor latency slowing, we calculated DMT, we used 1.0 ms as abnormal (we are not sure of the DMT magnitude). The DMT of 1.0 ms did reveal significant differences between the initially stratified groups but did not reach significance in outcome prediction modeling. More robust prospective studies of Edx parameters with surgical outcomes which establish the comparison threshold for the diagnosis of TTS need to be performed.

## Conclusion

The best predictors of improvement with TTS release were found in patients who had preoperative Edx TTS and tibial nerve plantar symptoms. Determining what factors best predict surgical outcome will require prospective studies and may require anatomical evaluation of the tibial nerve in the TT with other modalities.
